# An Integrated Methodology to Study Riparian Vegetation Dynamics: From Field Data to Impact Modeling

**DOI:** 10.1029/2020MS002094

**Published:** 2020-08-19

**Authors:** M. Latella, M. B. Bertagni, P. Vezza, C. Camporeale

**Affiliations:** ^1^ Department of Environmental, Land and Infrastructure Engineering Politecnico di Torino Turin Italy

**Keywords:** flow‐vegetation interactions, impact models, model calibration, riparian vegetation, stochastic processes

## Abstract

Riparian environments are highly dynamic ecosystems that support biodiversity and numerous services and that are conditioned by anthropogenic activities and climate change. In this work, we propose an integrated methodology that combines different research approaches—field studies and numerical and analytical modeling—in order to calibrate an ecohydrological stochastic model for riparian vegetation. The model yields vegetation biomass statistics and requires hydrological, topographical, and biological data as input. The biological parameters, namely, the carrying capacity and the flood‐related decay rate, are the target of the calibration as they are related to intrinsic features of vegetation and site‐specific environmental conditions. The calibration is here performed for two bars located within the riparian zone of the Cinca River (Spain). According to our results, the flood‐related decay rate has a spatial dependence that reflects the zonation of different plant species over the study site. The carrying capacity depends on the depth of the phreatic surface, and it is adequately described by a right‐skewed curve. The calibrated model well reproduces the actual biogeography of the Cinca riparian zone. The overall percentage absolute difference between the real and the computed biomass amounts to 9.3% and 3.3% for the two bars. The model is further used to predict the future evolution of riparian vegetation in a climate‐change scenario. The results show that the change of hydrological regime forecast by future climate projections may induce dramatic reduction of vegetation biomass and strongly modify the Cinca riparian biogeography.

## Key Points

- We combined remote sensing, field data, and numerical and stochastic modeling to study a riparian area
- Topography and water level fluctuations condition the long‐term distribution of riparian vegetation
- Spatial clusters based on the probability of inundation detect areas of homogeneous vegetation

## 1 Introduction

The riparian biome is an extremely dynamic ecotone, which connects aquatic and terrestrial ecosystems. It is one of the most diverse, complex, and active transitional habitats on Earth, supporting very high biodiversity (Naiman & Decamps, 1997; Tockner & Stanford, 2002). The interactions between aquatic and riparian ecosystems involve several physical and biotic processes. Riparian ecosystems retain water runoff and dampen river floods, improve water quality by acting as natural filters, and actively participate to river food webs and carbon sequestration (Allan & Castillo, 2007; Naiman et al., 2005). Furthermore, riparian areas have an important recreational and social value (Daily, 1997).

Nowadays, riparian ecosystems are facing rising anthropogenic threats, mainly due to human population growth and an increasing exploitation of water resources (Gordon et al., 2018; Vörösmarty et al., 2010). Additionally, freshwater ecosystems worldwide are struggling to cope with changing weather patterns (Jacobsen et al., 2012). To counteract these threats, river managers usually intervene with stream restoration after a negative biological indicator is detected, such as a loss of biodiversity or species population. However, it is a common feature of these negative trends to exhibit a strong resilience so that by the time they are detected, they cannot be readily reversed (Tonkin et al., 2019). In this scenario, it is crucial to develop models able to provide a deeper understanding of aquatic and riparian ecosystems, as well as accurate future predictions.

The difficulty in developing reliable models is due to the complex and interdependent processes that underlie the riparian ecotone dynamics. In fact, the temporal and spatial evolution of the riparian landscape is regulated by the mutual interactions among three major factors: (i) the hydrological fluctuations of the river flow, (ii) sediment transport dynamics, and (iii) the vegetation dynamics (Bertagni et al., 2018; D'Alpaos et al., 2016; Vargas‐Luna et al., 2015). In the field of ecomorphodynamics, the latter factor has been recently recognized as a key element in the evolution of the riparian landscape, since vegetation is not just a passive obstacle for the fluid flow but an active element in the whole riparian system where plants, sediments, and water continuously interact among them. See Vesipa et al. (2017) for a recent review.

Regarding the vegetation‐sediment feedbacks, dense vegetation reduces flow velocity and shear stress at the bed, thus decreasing the local sediment transport rate and promoting local sedimentation (Bennett et al., 2008; Neary et al., 2012). Plant roots and woody debris increase sediment cohesion, stabilizing banks and bars (Gurnell et al., 2001). The combination of deposition and stabilization promotes the accretion of the river bars and the generation of new space for plant colonization, which in turn will further favor these processes (Parker et al., 2011). Moreover, river braiding capacity has been shown to reduce with the increasing density of vegetation on the banks, as a higher plant coverage increases the sediment tensile strength, reduces bank migration, and favors narrower and deeper channels (Gran & Paola, 2001).

The vegetation‐hydrology feedbacks are of crucial importance, as every stage of plant life depends on water availability. In particular, the cycle of vegetation growth in riparian area is mainly determined by the occurrence of floods, which have both a constructive and a destructive role. Intense floods can cause scour, vegetation uprooting, and mechanical damage (Karrenberg et al., 2003). Yet they also disperse seeds and create bare moist sites rich in nutrients where the recruitment of new vegetation may take place (Mahoney & Rood, 1998; Shafroth et al., 2000). The successive growth from seedlings to saplings, and then to mature trees, successfully occurs for an optimal range of water levels (Camporeale & Ridolfi, 2006; Camporeale et al., 2013). Too dry conditions lead plants to suffer drought and die (Francis, 2007; Pasquale et al., 2012), while extremely moist soils prevent oxygen in reaching the roots, thus inducing anoxia (Vesipa et al., 2017).

Despite a strong influence of the deterministic trends of water levels on the growth and decay of the riparian biomass (e.g., seasonal snow melting or daily evapotranspiration cycles), the vegetation dynamics is crucially affected by the random fluctuations (noise) of water levels induced by stochastic rainfalls (Vesipa et al., 2017). The constructive role of noise in environmental phenomena has been widely recognized only recently. Noise may induce ordered states, periodic oscillations, or spatial patterns that would not exist in purely deterministic dynamic systems (D'Odorico et al., 2005; Ridolfi et al., 2011). For riparian ecosystems, the absence of water level fluctuations leads to one of the two deterministic stable states: fully vegetated or bare environment (Camporeale & Ridolfi, 2006, 2007; Vesipa et al., 2017). Conversely, when these fluctuations are significant, three different scenarios can occur, accordingly to the frequency and magnitude of the disturbance: (i) the riparian system fluctuates around one of the two states (bare and fully vegetated); (ii) the two states coexist in different time intervals; (iii) the features of the disturbances are such that the system exhibits a statistically stable intermediate state (Camporeale & Ridolfi, 2007; Camporeale et al., 2013).

In order to study the complex physical phenomena that characterize riparian environments, various approaches have been adopted, ranging from statistical studies of field data (Caponi et al., 2019; Edmaier et al., 2011; Edwards et al., 1999; Francis, 2007; Gurnell et al., 2001) and experimental setup (Gran & Paola, 2001; van Dijk et al., 2013) to analytical (Calvani et al., 2019; Camporeale & Ridolfi, 2006; Muneepeerakul et al., 2007) and numerical modeling (Bertoldi et al., 2014; Caponi & Siviglia, 2018; Lytle & Merritt, 2004; van Oorschot et al., 2016). In this paper, we combine most of the above‐mentioned approaches into an integrated methodology that provides closed relationships for riparian vegetation statistics, calibrated onto real data. The aim is to unite theoretical approaches and field analyses. The methodology is here applied and validated for the Cinca River (Spain), but it can be replicated to other rivers worldwide.

We use the minimalist stochastic model by Camporeale and Ridolfi (2006), which accounts for the fundamental physical and biologic processes involved in the riparian zone, as theoretical basis. For an ideal geometry, the same authors showed that the model can be solved in a purely analytical way. Instead, for real fluvial environments, the model requires a calibration supported by hydrodynamic simulations, field data collection, and GIS‐aided analyses. We encompass all these approaches in a unique methodology and calibrate the model for riparian vegetation dynamics referring to the real case of the Cinca River.

**Figure 1:**
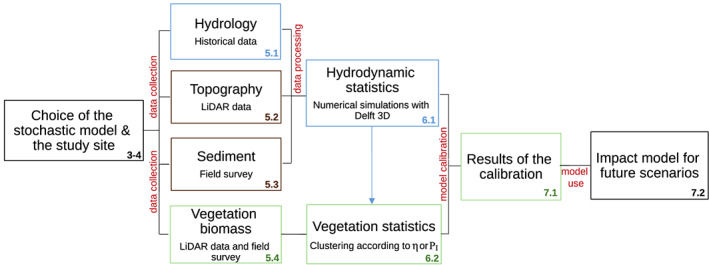
Workflow of the integrated methodology applied to calibrate the stochastic model for riparian vegetation dynamics. The calibrated model can be used to predict the impact of climate change on the riparian environment. Numbers refer to the sections in the paper.

The integrated methodology is a multistage process that involves several in‐series and parallel operations. We invite the reader to refer to Figure 1 and the next section as conceptual maps for the methodology and the paper outline.

## 2 Outline of the Integrated Methodology

We provide a conceptual map of the approach in Figure 1. The integrated methodology aims to calibrate a stochastic model for the vegetation dynamics. In the present study, we adopt the model by Camporeale and Ridolfi (2006), but the methodology can also be applied to other stochastic models (see section 3 for a brief review). This model provides the probability density function (pdf) of the above‐ground woody biomass once the topographical, hydrological, and biological data are known.

After the site identification (section 4), data collection is firstly organized through the hydrological characterization (section 5.1) and the analysis of LiDAR measurements for the topography and vegetation heights (section 5.2). The missing information about the grain size distribution (section 5.3) and the vegetation biomass (section 5.4) can be collected by performing a field survey.

The second step is the definition of the water level statistics by analyzing the outcomes of a two‐dimensional hydrodynamic modeling working on the flow rate time series (blue blocks in Figure 1, section 6.1). The setup is based on the processing of LiDAR data for the topography (Appendix A) and the data collected during the field survey for the granulometry (brown blocks in Figure 1, section 5.3). A general description of the hydrodynamic modeling is reported in section 6.1, and further technicalities are summarized in Appendix  B. The hydrodynamic statistics are then used to group different areas of the study site, where vegetation has experienced the same hydrodynamic forcing, into classes, as reported in section 6.2.

The third step regards the adoption of the stochastic model and is the core of the procedure since it provides the biomass computation (green block in Figure 1). An exhaustive description of the model is given in section 3. The original model requires three biological parameters as input (the nomenclature partially differs from the original paper by Camporeale & Ridolfi, 2006): (i) the vegetation growth rate *ω*, (ii) the local carrying capacity *β*, and (iii) the decay rate *α*. The adopted nomenclature is shown in Table C1. The growth rate *ω* is related to the inverse of the timescale of plant growth *T*_*g*_. For instance, it may be defined as *ω* = 5.88/*T*_*g*_ by assuming a classical logistic equation for plant growth and by considering *T*_*g*_ as the time needed by a plant to undergo a growth of the 90% of its maximum size, namely, from 5% to 95% (Camporeale & Ridolfi, 2006; Perucca et al., 2009). Significative *ω* values for some of the most common riparian species are reported in Table 1. The carrying capacity *β* is often defined through an optimal function of the depth of the phreatic surface bounded by the conditions of anoxia and drought (Camporeale et al., 2013; Pearlstine et al., 1985; Phipps, 1979), but the coefficients of this function should be derived from site‐specific field measurements. The decay rate *α* parameterizes the decline of vegetation biomass during hydrodynamic stress conditions, being site dependent and strongly related to the root systems. Due to the uncertainty and variability in the literature values of these latter two parameters, the decay rate and the local carrying capacity become the goal of the model calibration, whose results are shown in section 7.1. Differently, as the calibration only requires the steady‐state solution of the biomass distribution, a time rescaling actually allows the following analysis to be independent of the value of the growth rate.

**Table 1:** Growth Rate *ω* of the Most Common Species in Riparian Environments

Tree species	Common name	*ω* (10^−5^ d^−1^)
*Carya tomentosa*	Mockernut hickory	6.5^a^
*Liriodendron tulipifera*	Yellow poplar	8.0^a^
*Nyssa aquatica*	Water tupelo	6.5^a^
*Populus alba*	Silver poplar	5.7^c^–12^b^
*Populus deltoides*	Eastern cottonwood	13^a^
*Populus nigra*	Black poplar	15^b^–23^c^
*Prunus serotina*	Wild cherry	7.8^a^
*Salix alba*	White willow	23^b^–77^c^
*Salix nigra*	Black willow	28^a^

a Camporeale and Ridolfi (2006).

b San‐Miguel‐Ayanz et al. (2016).

c Leuschner and Meier (2018).

Finally, the present methodology is adopted in section 7.2 to build a local impact model (LIM) for the study site. To this purpose, we set a new hydrological scenario, in agreement with the climate projections within 2100 (Alfieri et al., 2015), and we forecast the vegetation response to the new forcing.

## 3 Stochastic Model

To model the vegetation dynamics, we adopt a minimalist ecohydrological approach that grounds on the fundamental processes occurring in riparian environments, still keeping the analytical solvability of the model equations. Although some simplifications are required, the so‐called low‐order models have provided successful results in several branches of the biogeosciences (Ridolfi et al., 2011), such as vegetation pattern formation (D'Odorico et al., 2006; Rietkerk & Van de Koppel, 2008) and ecohydrology (D'Odorico et al., 2005; Rodrigez‐Iturbe & Porporato, 2005). Stemming from the work by Camporeale and Ridolfi (2006)—henceforth referred to as CR06—stochastic approaches have also been proposed to describe the riparian vegetation ecosystems (Camporeale & Ridolfi, 2007, 2010; Crouzy & Perona, 2012; Muneepeerakul et al., 2007; Perona, Camporeale et al., 2009; Perona, Molnar et al.,^2009^; Perucca et al., 2009; Tealdi et al., 2010, 2013). For simplicity, we refer to CR06, but other schemes can also be considered. The salient aspects of the model are summarized in the following (further details can be found in the original publication).

CR06 accounts for two major factors affecting vegetation growth and decay, namely, the topography and the randomness of water stage fluctuations. As output, CR06 provides analytical solutions for the steady‐state pdf of the above‐ground woody biomass and its statistical moments. The main assumptions are (i) the interspecies interactions due to synergy or competition are overlooked, although the features of the various species are taken in account to set the vegetation‐related parameters; (ii) a steady river morphology is considered, neglecting sedimentation, erosion, and feedback between topography and vegetation; and (iii) the time delay between the vertical oscillations of river water stage and the related change in the groundwater level in the adjacent unconfined aquifer is negligible, if compared to the timescale of interactions between vegetation and groundwater.

**Figure 2:**
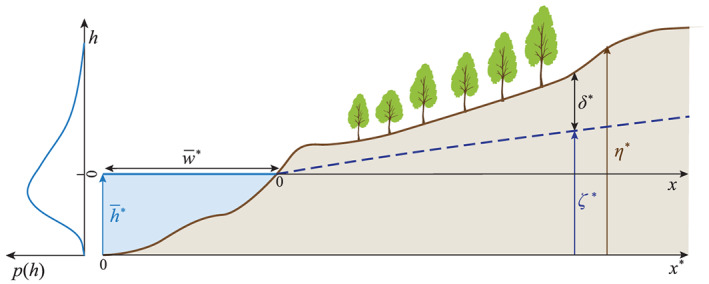
Sketch of the riparian transect where ${\bar{h}}^{*}$ is an average free‐surface water stage. A qualitative behavior for the free‐surface pdf *p*(*h*) is reported on the left.

According to CR06, the stochastic dynamics of vegetation are mainly affected by the topography and the temporal randomness of the flow. Geometrically speaking, the fluvial reach is identified as a generic riparian transect with the origin of the vertical axis coincident with the minimum value of water surface (Figure 2). This configuration is completely described by the topographic elevation *η*^∗^(*x*) and the phreatic water stage *ζ*^∗^(*x*,*h*), while *δ*^∗^(*x*,*h*)=*η*^∗^(*x*)−*ζ*^∗^(*x*,*h*) defines the depth of the phreatic surface. Water level fluctuations are represented by the random variable *h*^∗^ that is described by its pdf *p*(*h*^∗^) and its integral scale *τ*^∗^, which is the area of the autocorrelation function, namely, a proxy of the process memory (Cox & Miller, 2001). To simplify the notation, the mean water level ${\bar{h}}^{*}$ and the transect width ${\bar{w}}^{*}$ are adopted to scale the main variables

(1) $$h\;=\;\frac{h^{*}-{\bar{h}}^{*}}{{\bar{h}}^{*}}\;\eta \;=\;\frac{\eta^{*}-{\bar{h}}^{*}}{{\bar{h}}^{*}}\;\zeta \;=\;\frac{\zeta^{*}-{\bar{h}}^{*}}{{\bar{h}}^{*}}\;x\;=\;\frac{x^{*}-{\bar{w}}^{*}}{{\bar{w}}^{*}}.$$

Vegetation features are summarized by the dimensionless local carrying capacity *β*, the decay rate *α*, and the growth rate *ω*. The local carrying capacity characterizes the maximum size that the vegetation can reach in local optimal conditions (i.e., high nutrient availability and optimal soil moisture), whereas the decay rate accounts for the magnitude of flood‐related decrease of biomass. We emphasize that both parameters are site dependent.

Supported by numerical simulations, CR06 showed that, if time is rescaled with the growth rate (*t* = *ωt*^∗^), the decay and growth of biomass *ν* in response to the stochastic hydrology are described by the following dichotomic process

(2) $$\frac{d\nu }{dt}\;=\;\left\{\begin{array}{ll} -\alpha \nu^{n} & \;\text{(Inundation},h\geq \eta \text{)}\\ \nu^{m}{\left(\beta -\nu \right)}^{p} & \;\text{(Exposure},h<\eta \text{)}, \end{array}\right)$$

where *m*, *n*, and *p* are numeric constants depending on the features of the vegetation. The decay factor *α* is defined as

(3) $$\alpha (k)\;=\;\frac{k}{P_{I}}\int_{\eta }^{\infty }(h-\eta )p(h)dh,$$

where *k* is a decay coefficient related to the features of the vegetation and *P*_*I*_ is the weighted average inundation probability. The dichotomic stochastic model (2) can be solved in terms of the asymptotic steady‐state probability distribution function *p*(*ν*)

(4) $$p(\nu )\;=\;\frac{N}{\alpha }\nu^{\frac{\beta (1-\alpha \tau )-(\alpha +\beta )P_{I}}{\alpha \beta \tau }}{\left(\beta -\nu \right)}^{P_{I}/\beta \tau -1}(\alpha +\beta -\nu ),$$

where *N* is a normalization constant. Equation 4 is a central result of CR06, and it is valid provided

(5) $$P_{I}<\beta /(\alpha +\beta ),$$

otherwise *p*(*ν*) is a Dirac's delta in *ν* = 0. Since *α* depends on the bed elevation *η*(*x*) through the Equation 3 and the quantity *β* is a still unknown site‐dependent parameter, Equation 5 also states the physical limits of the riparian corridors.

All the statistical moments of the random variable *ν* may be readily computed from Equation 4. We remark that, because the process is statistically stationary and has a finite integral scale, one may interpret these moments as temporal averages (Slutsky's Ergodic Theorem). Yet their actual definition is related to ensemble statistics. Indeed, as explained in the following, we performed the averaging of the biomass density over several sites experiencing the same hydrodynamic forcing that is basically an ensemble averaging of different realizations of the same process.

We also highlight that a closed‐form computation of *α* is only allowed for very simplified cross‐sectional geometries. In contrast, in real cases, the identification of a site‐dependent rating curve which relates discharges to water levels is a necessary condition that requires the adoption of a two‐dimensional hydrodynamic modeling over the study area. This delicate procedure is the ultimate aim of section 6.

## 4 Study Site

**Figure 3:**
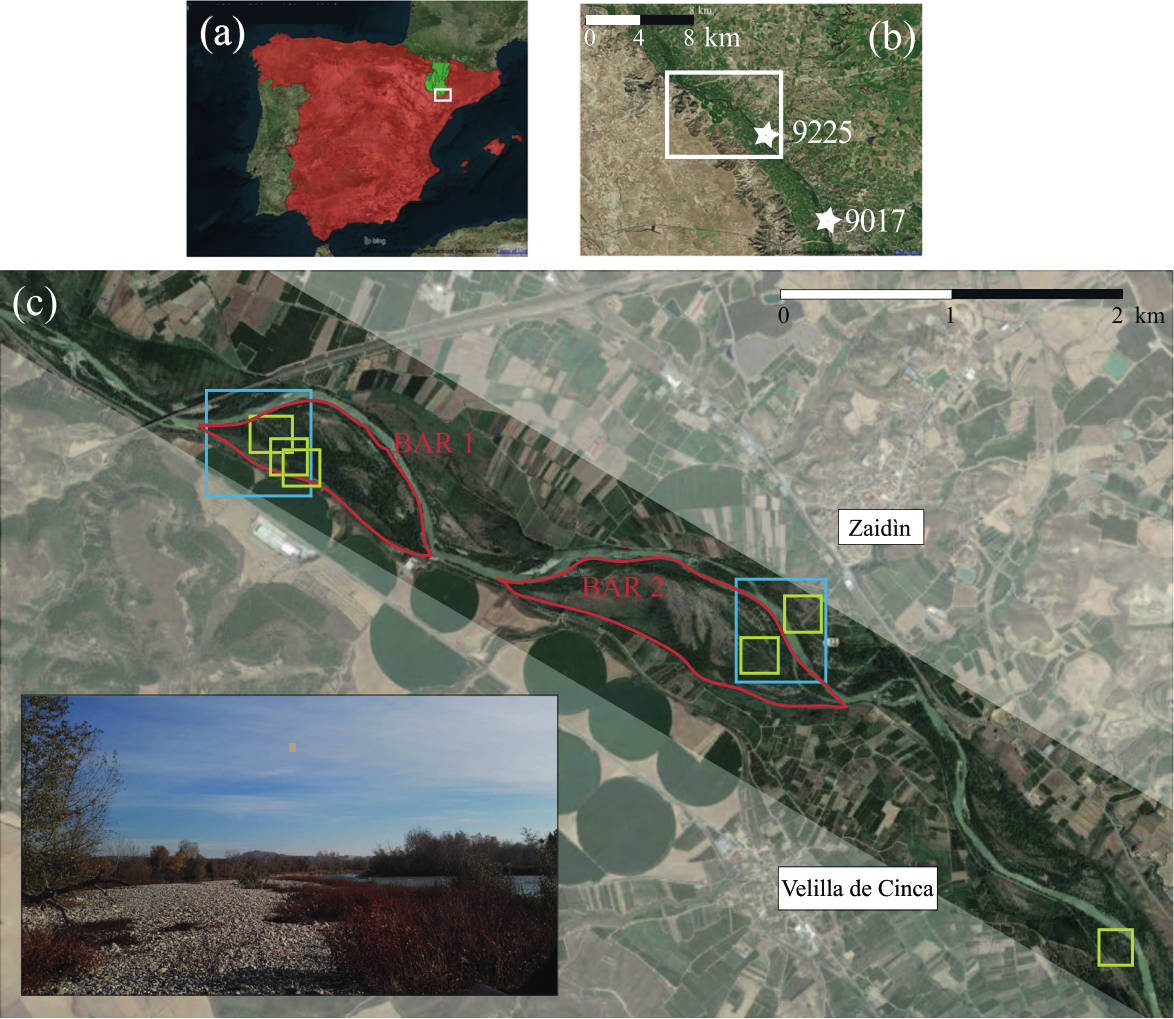
The study site from (a) country to (c) local scale. Every white rectangle indicates the content of the subsequent panel. (a) Catchment of the Cinca River in the northeastern Spain. (b) The location of the two gauging stations of the Ebro measuring network (stars). (c) The red lines are the contours of the modeled bars. Eight subareas were investigated during a field survey, which included sediment sampling (light‐blue boxes) and vegetation measuring (green boxes). The picture was taken by the authors during the field survey.

The study site is a segment of the Cinca River in the Spanish region of Aragón (Figure 3). The Cinca River is a tributary of the Segre River. The confluence Cinca‐Segre is located at La Granja d'Escarp, not far from the point where the Segre River flows into the Ebro River. The Cinca River is 191 km long and has a 10,000 km^2^ catchment area. It springs in the Pineta Valley, within Pireneos Mountains, and it flows from NE toward SW, ending in the confluence with the Segre River.

The selected segment is bounded upstream at the confluence with the Alcanadre River, close to the town of Ballobar, and downstream at the confluence with the Clamor Amarga River, close to the town of Zaidín. The length of the selected segment is approximately 9 km, and the corresponding catchment area is around 9,400 km^2^. The Cinca River is characterized by a nivo‐pluvial regime, showing two maxima in late spring and autumn. Its mean flow rate is approximately 73 m^3^ s^−1^, whereas the flow rates corresponding to return time equal to 5, 10, and 100 years are 126, 169, and 304 m^3^ s^−1^, respectively.

The site was chosen for the spatial variability in the morphology, its negligible topographic change through time, and the availability of a LiDAR data set (dated 2016) and hydrological data over a long period. In addition, the degree of anthropogenic pressure is moderate, mainly due to the presence of a road connecting the two small towns of Ballobar and Zaidín and some field crops.

**Figure 4:**
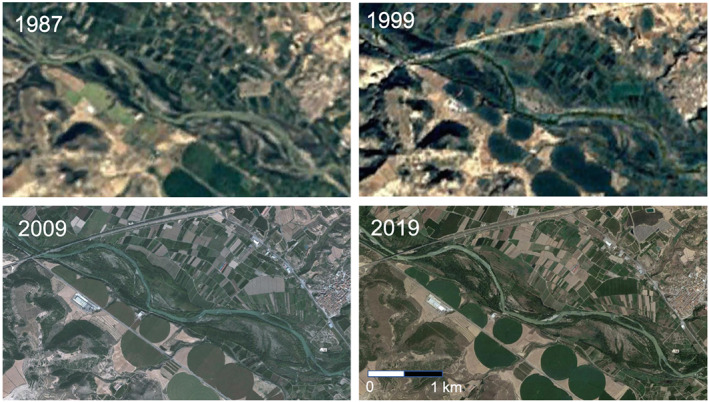
Satellite images showing that the two bars have been morphologically stable for several decades (source: Google Earth).

In the considered reach, the river is wandering with moderately vegetated bars and islands. The braiding index related to the active channel (i.e., the mean number of the active separated channels per each cross section) is lower than 1.5 (Rinaldi et al., 2015). The floodplain, more than 2 km wide, is bounded by Quaternary terraces. As shown in Figure 4, no significant morphological changes has occurred over the last 40 years within the study site, except for a weak river incision, so that vegetation growth has only been influenced by water level fluctuations. This site is therefore suitable for the application of the reference model that, as explained in section 3, assumes a steady morphology.

The riparian vegetation mainly grows either in a narrow band along the river banks or above islands and fluvial bars because of the pressure of crop fields that usually occupy most of the floodplain. Most of the vegetation is constituted by shrubs and trees, whose dominant species are willows (*Salix elaeagnos*, *Salix atrocinerea*, and *Salix alba*), poplars (*Populus nigra* and *Populus alba*), and some mastics (*Pistacia lentiscus*). Grass‐like plants, such as common reeds (*Arundo donax* and *Phragmites australis*) and bulrush (*Typha sp*.), grow where inundations are frequent. The vegetation cover is quite homogeneous over the river bars, except for some areas characterized by a bare soil or a less developed plants.

## 5 Data Collection

With the purpose of applying the present methodology to the Cinca River, we collected all the input data necessary for the stochastic model.

Streamflow time series and LiDAR data, provided by local authorities (CHE, 2010; IGN, 2015), were used to estimate: (i) the hydrological characteristics of the flow regime, (ii) the topography of the riverbed, and (iii) the vegetation heights. In addition, we carried out a field survey on 26–28 November 2018 to assess the grain size distribution, which is fundamental to define the hydraulic roughness and the vegetation allometric relationships that were used to estimate the biomass. The hydraulic roughness and the vegetation features are mandatory information for the two‐dimensional hydrodynamic modeling.

### 5.1 Hydrology

Hydrometric data were provided by the network of gauging station of the Ebro River Basin authority (CHE, 2010). Data were taken from the station 9017, close to the town of Fraga. A few kilometers upstream of the same station, the Clamor Amarga River flows into the Cinca River, providing 6.5% of the annual flow rate. Therefore, the Clamor Amarga River negligibly contributes to the total streamflow, and the hydrometric data collected by station 9017 can be considered as representative of the selected river segment.

**Figure 5:**
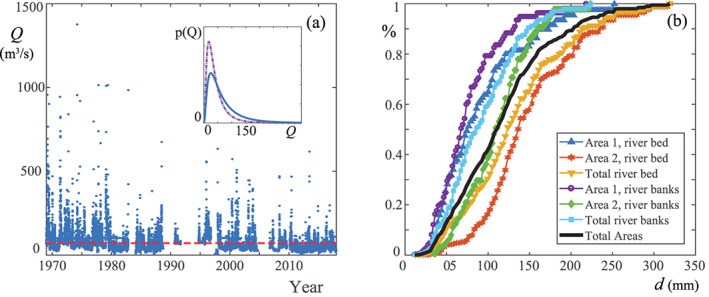
Flow rate and sediment data. (a) Daily flow rates *Q* of the Cinca River (gauging station 9017) and related pdf (in the inset). The red dotted line indicates the average flow rate ( $\bar{Q}$ = 71 m^3^ s^−1^). The dash‐dot line in the inset is the forecast pdf of the discharge in a climate‐change scenario (see section 7.2). (b) Grain size distribution for the various sampling areas (% is the relative frequency for the various diameter *d* classes). The black line comprises the samples collected in all areas.

The whole available daily flow rates time series ranges from 1947 to 2017 (Figure 5a). However, the El Grado reservoir was built upstream the study area in 1969, inducing a flow regulation. Therefore, in order to grant the stationarity of the time series, we only considered the data after 1969.

### 5.2 Topography

A digital terrain model (DTM) was obtained by processing a free LiDAR data set provided by the Spanish National Center of Geographic Information (IGN, 2015). The study area was scanned by IGN on 20 October 2016 by using a small‐footprint discrete‐return airborne laser scanner ALS60. The wavelength, laser pulse rate, and nominal mean return are 1,064 nm, 45 kHz, and 0.5 points m^−2^, respectively. The geodetic reference system is ETRS89, and the cartographic projection is UTM. The standard deviation of elevation is lower than 0.20 m.

The LiDAR data set was processed through the software FUSION/LDV in order to have a DTM of the site and the statistics about vegetation height and cover (i.e., the percentage of the soil area covered by the projection to the ground of tree canopies). These statistics allowed for the biomass estimation, as explained in section  5.4. Further technical details about LiDAR data processing are provided in Appendix A.

### 5.3 Sediment and Roughness Characterization

The bed of the Cinca River is composed by coarse sediments (conglomerates and gravel) and a fine fraction of clay (Confederación Hidrográfica del Ebro, 2002a, 2002b). The grain size data were collected during the field survey and then processed in order to properly quantify the roughness of Cinca's riverbed. Samples were collected in two areas (blue boxes in Figure 3c), within which both the river bed and its banks were investigated.

The sediment analysis was performed by combining the *Wolman Pebble Count* method and the BaseGrain software. The Wolman Pebble Count consists of random sampling along a zig‐zag trajectory, including both the river banks and the bed (Harrelson et al., 1994; Leopold et al., 1964). BaseGrain is a MATLAB‐based image‐processing tool that allows for the identification of the largest and intermediate diameters of grains by using the contrast between the lighter grains and the darker background (Detert & Weitbrecht, 2012, 2013). The software output is a population of objects characterized by the intermediate diameter. Starting from this output, the grain size distribution can be obtained with (Detert & Weitbrecht, 2012)

(6) $$s_{i}\;=\;\frac{S_{i}{\left\langle d_{i}\right\rangle }^{0.8}}{\overset{n}{\underset{i=1}{\sum }}S_{i}{\left\langle d_{i}\right\rangle }^{0.8}},$$

where *s*_*i*_ is the ratio between the weight of fraction *i* and the weight of the entire sample, *S*_*i*_ is the ratio between the number of samples in fraction *i* and the number of total samples, ⟨*d*_*i*_⟩ is the mean diameter of fraction *i*, and *n* is the amount of fractions.

In our case, the collected samples were grouped over a dark fabric in order to obtain a high contrast picture, which improved the input for BaseGrain. This process was repeated 10 times within the two sampling areas, allowing for the size determination of 520 samples. The grain size distributions were then obtained for the individual sampling areas and for the whole site (Figure 5b). Accordingly, the values of *d*_50_ and the *d*_90_ were extracted and used to estimate the Manning coefficient *n*_*m*_ (see a sample of empirical formulas reported in Table 2), whose average value was estimated to be *n*_*m*_ = 0.03, in agreement with the previous literature (Confederación Hidrográfica del Ebro, 2002a; Murillo et al., 2008). The local roughness in the hydrodynamic modeling was then computed as the sum of the roughness of the bare soil and the roughness induced by plants (see also Appendix B).

**Table 2:** Estimation of the Manning Coefficient *n* for the Main Channel, the Bars, and the Whole Study Site

		Main channel			Bars			
Relation	*n*_*m*_	Area_1_	Area_2_	Total	Area_1_	Area_2_	Total	Study site
*d* _50(mm)_		74.46	136.34	123.95	67.78	109.17	86.37	111.11
*d* _90(mm)_		161.07	235.43	213.75	130.53	160.71	146.40	198.00
F. H. Adm.^a^	0.0482 $d_{50}^{1/6}$	0.0313	0.0346	0.0340	0.0308	0.0333	0.0320	0.0334
Julien^b^	0.062 $d_{50}^{1/6}$	0.0402	0.0445	0.0438	0.0396	0.0429	0.0412	0.0430
Strickler^c^	$d_{50}^{1/6}$/21.1	0.0307	0.0340	0.0335	0.0303	0.0328	0.0315	0.0329
Julien^b^	0.038 $d_{90}^{1/6}$	0.0280	0.0299	0.0294	0.0271	0.0280	0.0276	0.0290
M.‐P. and M.^d^	$d_{90}^{1/6}$/26	0.0284	0.0302	0.0297	0.0274	0.0284	0.0279	0.0294
Strickler^c^	0.036 $d_{90}^{1/6}$	0.0266	0.0283	0.0278	0.0256	0.0256	0.0261	0.0275
Average *n*_*m*_		0.0309	0.0336	0.0330	0.0301	0.0320	0.0311	0.0325

*Note*. For the numerical simulations, the average values (last row of the table) are used.

a FHWA (1979).

b Julien (2018).

c Strickler (1923).

d Meyer‐Peter and Müller (1948).

### 5.4 Vegetation Biomass and Allometric Relationships

By processing the raw LiDAR data (see Appendix A), we derived the statistics of vegetation height and cover, that is, the percentage of the soil area covered by the projection to the ground of tree canopies. We then linked these statistics to the above‐ground vegetation biomass density *V*—needed in the CR06 model—through the so‐called Da Vinci's rule (Tealdi et al., 2010),

(7) $$V\;=\;\frac{\lambda \rho H\pi D^{2}}{4}\;[{\mathrm{Mg}\;m}^{-2}],$$

where *ρ* is the fresh wood density, *H* is the tree height, *D* is the *diameter at breast height* (DBH), which is conventionally measured at 1.4 m from the ground, and *λ* is the number of tree stems per square meter. According to Equation 7, the sum of the cross‐sectional areas of branches at a fixed height is equal to the cross‐sectional area of the trunk (Eloy, 2011). Although this formula neglects the foliage biomass, it can be used for riparian species where foliage constitutes just 2–6% of the total biomass (Freedman et al., 1982; Jenkins et al., 2003; Ker, 1980).

Equation 7 requires the stem density *λ* and an allometric relationship between the vegetation height and diameter, namely, the function *D* = *D*(*H*). Several LiDAR‐based models have been regressed to this aim (Jenkins et al., 2003; Ter‐Mikaelina & Korzukhin, 1997), but most of them refer to different climates or different LiDAR technologies, as such we avoided their implementation in our study site (i.e., temperate climate and a discrete‐return and small‐footprint LiDAR equipment). Moreover, the few models referring to similar conditions (He et al., 2013; Li et al., 2008; Means et al., 2000) provide remarkably different results between each other, likely because of the diverse return densities (García et al., 2010; Mitchell et al., 2012; Wulder et al., 2012) and site dependencies (Jenkins et al., 2003; Ter‐Mikaelina & Korzukhin, 1997), thus preventing the selection of the most adequate relationship for our study case.

Therefore, in order to properly address the function *D*(*H*), we collected field data on the vegetation features within plots located in six areas of the study site (green boxes in Figure 3c). These were chosen according to site accessibility, as large part of the Cinca River banks and bars is colonized by thick brambles. Within the sampling plots, we surveyed the heights and DBHs of the main tree species, namely, willows and poplars, through a laser rangefinder (Trupulse 360R, Laser Technology, Centennial, Colorado, USA) and metric tapes. In addition, we estimated the tree density by a census of the tree population in areas of known extension, with an average value of *λ*=0.2 m^−2^. This value is consistent with previous studies in riparian areas (Rodríguez‐González et al., 2010; van Oorschot et al., 2016). The timing of the field campaign did not affect our vegetation measurements as follows: (i) we were not interested in the grass‐like plants that grow with annual life cycles; (ii) our measurements were not affected by the presence of tree foliage.

Three allometric relationships were regressed by the field data, both considering single species, respectively poplars and willows, and the mixed tree population,

(8) $$D(H)=\left\{\begin{array}{l} 0.005680\;H^{1.7337}\;\text{poplars}\\ 0.003345\;H^{1.6807}\;\text{willows}\\ 0.004312\;H^{1.7784}\;\text{mixed population} \end{array}.\right)$$

**Figure 6:**
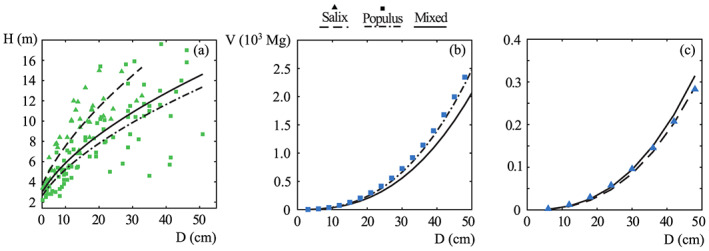
Vegetation height *H* and biomass density *V* versus the diameter at breast height D. (a) Allometric relationships from Equation 8 (black curves) regressed on the field data (green symbols). (b and c) Comparison between data from Young et al. (1980) (blue symbols) and Equation 7 for poplars and salix, respectively.

Figure 6a compares the field data with the regressed allometric relationships (*R*^2^ = 0.69, 0.86, and 0.68, respectively), while Figures 6b and 6c shows that Equations 7 and 8 well compare with literature data for the simple case of a single tree (Young et al., 1980). Fresh wood density was set to 0.950, 0.500, and 0.725 Mg m^−3^ for poplars, willows and the mixed population, respectively (Wangaard, 1979). In addition, we notice that a computation of the maximal areal biomass density for the study area, through Equation 7 with *λ* = 0.2 m^−2^, provides a value around 4,300 Mg ha^−1^, that is close to the value of 5,000 Mg ha^−1^ reported by Young et al. (1980) for an area characterized by humid continental climate.

By using the vegetation heights derived from the LiDAR data into Equations 7 and 8, we eventually obtained the areal above‐ground biomass density for each point of the study site. The plants lower than 1.4 m—shrubs, seedlings, and saplings—were excluded from the analysis.

## 6 Data Processing

### 6.1 Hydrodynamic Modeling

**Figure 7:**
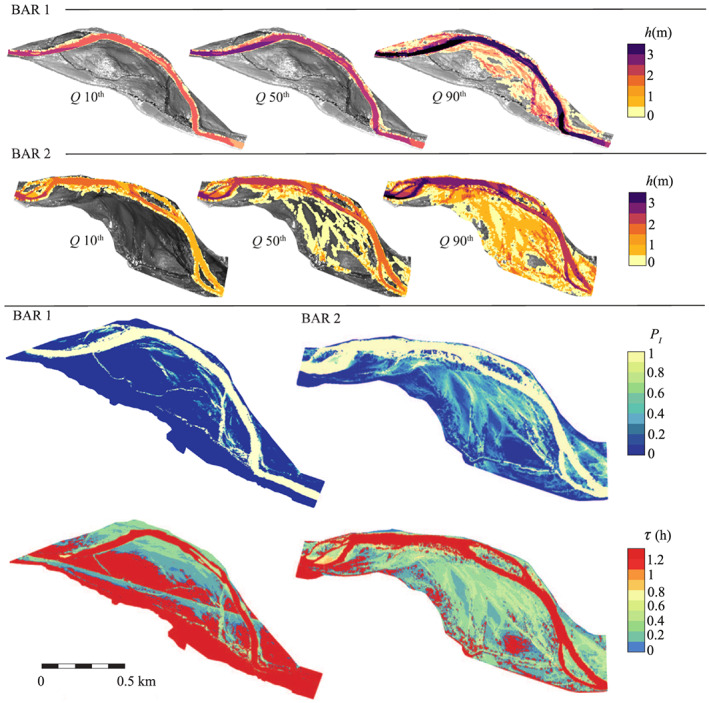
Hydrodynamic modeling for the two bars. The first two rows show the water depth distributions for three different flow rates, corresponding to the 10th (*Q* = 25 m^3^ s^−1^), 50th (*Q* = 52 m^3^ s^−1^) and 90th (*Q* = 135 m^3^ s^−1^). The third and fourth rows show the distribution of computed hydrological statistics, respectively the probabilities of inundation *P*_*I*_, from Equation 9, and the integral scales *τ*, from Equation 10.

As explained in the description of the stochastic model (section 3), the analytical evaluation of the necessary hydrological statistics (inundation probabibility *P*_*I*_ and integral timescale *τ*) is precluded by the topographic and hydraulic complexity of the site. Consequently, we developed a two‐dimensional hydrodynamic modeling in the domain located over the two fluvial bars (red contours in Figure 3c) in order to relate the discharge time series to the necessary hydrodynamic statistics. The results of the procedure are reported in Figure 7 for the two bars. We discretized the pdf of discharge in 19 quantiles, and for each of them, we launched a two‐dimensional hydrodynamic simulation by using the latest *Flexible‐Mesh* version of the software Delft3D (Appendix B), which also accounts for the hydraulic resistance induced by vegetation (i.e., *trachytope*). As a result, the 2D flow field and the water levels over the study domain were obtained for each flow rate, allowing the rating curve (*Q*‐*h* relationships) to be defined for each computational cell. In addition, from the discharge time series (Figure 5a), a pdf for the water stage *p*(*h*) was derived in each computational cell so that the probability of inundation *P*_*I*_ was calculated according to

(9) $$P_{I}\;=\;\int_{\eta (x)}^{\infty }p(h)dh.$$

Likewise, by computing the mean duration time of exposure *τ*_*E*_ and inundation *τ*_*I*_, we also got the integral scale of the dichotomic process (Ridolfi et al., 2011) for each vegetation class (see section 6.2) as

(10) $$\frac{1}{\tau }\;=\;\frac{1}{\tau_{E}}+\frac{1}{\tau_{I}}.$$

### 6.2 Site Discretization

**Figure 8:**
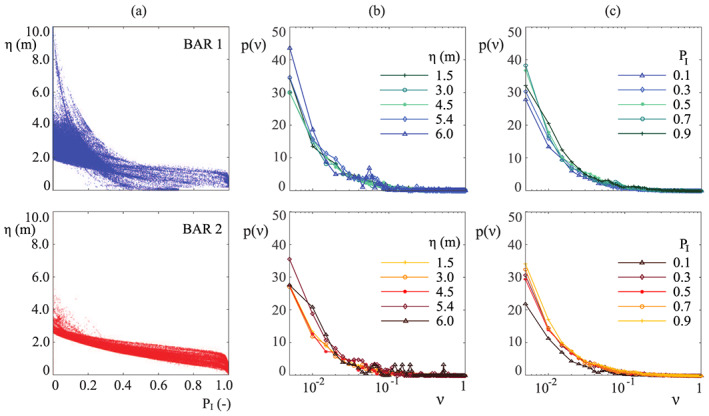
Vegetation data analysis and classification. (a) Relation between the elevation *η* and the probability of inundation *P*_*I*_ for the two bars. (b) Real pdfs of vegetation computed according to the *η* classification. (c) Real pdfs of vegetation computed according to the *P*_*I*_ classification. Upper row: bar 1; lower row: bar 2.

The model CR06 eventually provides vegetation statistics as a function of the hydrodynamic forcing that influence plant growth, that is, water availability, uprooting and mechanical damages. Therefore, we divided the study site into classes, defined in order to collect the plots that experienced the same hydrodynamic forcing. Two criteria were adopted for the class definition: (i) the elevation and (ii) the inundation probability. The first classification (hereafter referred to as *η* classification) considers the topographic height *η* to be the main factor influencing plant growth, and it is very common in the literature when referring to the riparian zone (Malanson, 1993; Naiman et al., 2005). The second classification is based on the inundation probability, arbitrarily subdivided into intervals of Δ*P*_*I*_ = 0.1 (*P*_*I*_ classification). The two criteria are expected to provide similar results just under very simplified topographic conditions, when there is a deterministic relation between inundation and topography, and to differ when the *η*‐*P*_*I*_ relationship is scattered. As Figure 8a shows, the *η*‐*P*_*I*_ relationship is more scattered for bar 2 than bar 1, as a result of the complex interactions between water flow and bar topography. Consequently, *η* and *P*_*I*_ classifications are expected to provide different results, especially for the bar 1.

The real pdfs of the biomass density distributions are shown for each class in Figures 8b and 8c. The *η* classified real pdfs show a peak for *ν*→0, related to the smallest plants (after the exclusion of vegetation lower than 1.4 m) and then exponentially decrease at large values of *ν*. The peak value increases with *η*, thus indicating that the presence of higher vegetation decreases for increasing altitudes. This trend is in agreement with our observations during the field survey, and it is likely related to the effects of deep water tables at higher elevations. It suggests that soil moisture availability is the dominant limiting factor for vegetation growth in the present system. We also notice that the *η* classified pdfs are not unimodal, since they have unpredictable peaks at random *ν* values. This denotes the presence of nonhomogeneous groups of trees within the same class, and it is a clue of weakness of this kind of classification. Indeed, these irregular patterns may be related to areas with different inundation probabilities within the same topographic class.

Although the *P*_*I*_ classified pdfs show similar exponential trends (Figure 8c), irregular patterns are rarer and smaller than in the *η* classification so that the pdfs are generally smoother. This implies a more homogeneous behavior of vegetation features within each class and suggests that the *P*_*I*_ classification is the best option for the model calibration. However, for comparison purposes, the model calibration is here illustrated for both criteria.

## 7 Model Calibration and Use

We here implemented the stochastic model by Camporeale and Ridolfi (2006) for the real case study of the Cinca River, aiming to provide a calibrated model suitable for future forecasting of vegetation response to external forcing. The CR06 model gives the pdfs of the dimensionless vegetation biomass from the hydrological, topographical, and biological data. The biological parameters are the growth rate *ω*, the decay rate *α*, and the local carrying capacity *β* (see section 3). As aforementioned in section 2, the growth rate *ω*, whose typical values for some riparian species are shown in Table 1, plays just the role of a scaling parameter, which does not affect the results of the calibration, since we adopted a steady‐state solution of the stochastic model. The local carrying capacity *β* is usually modeled as a bell‐shape or parabolic function of the water table depth (Gónzalez et al., 2012; Phipps, 1979). However, the characterization of these curves is usually site dependent and affected by local disturbances, and we, therefore, opted for a least‐square minimization procedure onto real data. The decay rate *α* is also very site specific and still an open issue in the literature (Muneepeerakul et al., 2007). From Equation 3, *α*(*k*) is computed as *k* times an integral quantity depending on the elevation and hydrology of the site, where *k* is related to the intrinsic features of the vegetation. The numerical hydrodynamic modeling allows the integral term to be defined (see section 6.1), but *k* still remains unknown and it thus becomes the second parameter to be calibrated.

### 7.1 Calibration Results

During the calibration phase, the whole study site was divided in cells, successively ranked according to the two classification criteria (section 6.2). Hydrological and topographic parameters were then computed for each class, and a set of various {*k*,*β*} pairs were considered iteratively. At each iteration, the theoretical pdf of vegetation biomass *p*(*k*,*β*) was computed for every class through Equation (4) and compared with the real one. The final values of *k* and *β* were provided by the best fitting through a least‐square minimization between the real and theoretical pdfs. Notice that *β* ranges from 0 to 1, whereas *α*(*k*) has an upper threshold given by Equation 5.

**Figure 9:**
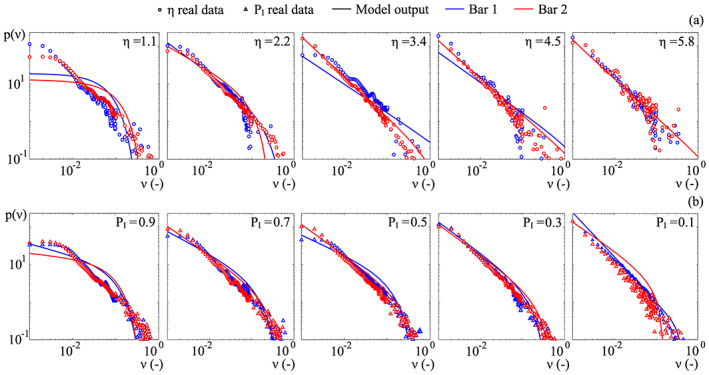
Pdfs of the dimensionless vegetation biomass. The comparison is carried out between the field data (symbols) and the theory (solid line) for the *η* (a) and the *P*_*I*_ (b) classification.

Figure 9 compares the theoretical pdfs obtained by means of the calibration with the real ones. The *P*_*I*_ classification fits quite well the real pdfs (triangles), especially for *P*_*I*_<0.9, that is, where inundation is not too frequent. Conversely, the *η* classification shows a poorer fit on the real pdfs (circles). In particular, the *η* classification performs worse at the highest elevations for the first bar (*η*>4.3), as the carrying capacity *β* drops to zero. For both the classifications, the fit is poor for those sites that are submerged for most of the time (*P*_*I*_ = 0.9 and *η* = 1.1 in Figure 9). Likely, these sites are affected by condition (5), according to which when *P*_*I*_ approaches 1 (always submerged), the upper threshold of *α*(*k*) tends to zero, thus reducing the range of possible values of *k* for the best fitting procedure.

**Figure 10:**
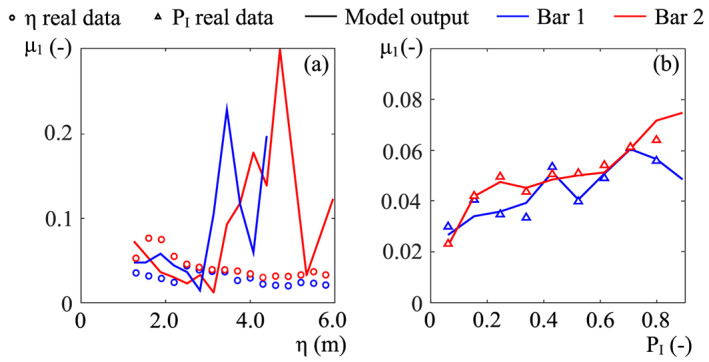
Mean value of the dimensionless vegetation biomass. Comparison among the field data (symbols) and the theory (solid line) for the *η* (a) and the *P*_*I*_ (b) classification. It emerges the superiority of *P*_*I*_ classification in reproducing the average vegetation biomass.

The first moment of the pdfs (i.e., the average value) is reported in Figure 10 for both the classifications. We see that the *P*_*I*_ classification (panel *b*) is again superior than *η* classification (panel *a*). The former is in fact able to catch a positive correlation between the inundation rate and biomass, likely related to species zonation (see later). In the *η* classification, it seems instead that a very mixed population within each class generates a constant mean value that is hardly fitted by the theoretical model. These differences are related to the irregular patterns appearing in the *η* classification, which jeopardize the correct evaluation of the statistical moments. It is worth noticing that the average vegetation biomass increases with the probability of inundation, meaning that flooding is not the major control factor in the site, and highlighting the crucial role played by the distance from the water table.

**Figure 11:**
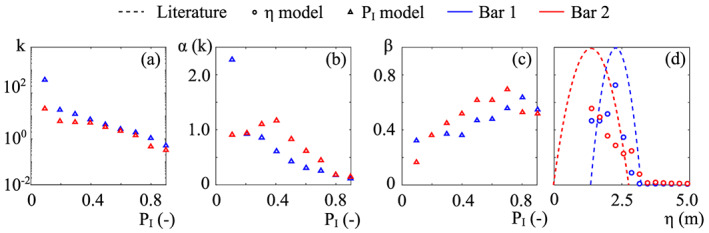
Results of the calibration for the (a) decay coefficient *k*, (b) the decay rate *α*, and (c) carrying capacity *β*.(d) Comparison between calibrated *β* values and the parabolic shape suggested in the literature (dashed lines).

In Figure 11, we reported the values of *k*—for *α*(*k*)—and *β* resulting from the calibration procedure. In previous works, the decay coefficient *k* has arbitrarily been set as a constant value, ranging from 2.5 to 10 (Camporeale & Ridolfi, 2006; Muneepeerakul et al., 2007). However, the present field‐based calibration provides a decreasing exponential decay between *k* and *P*_*I*_ (Figure 11a). The trend is very similar for the two bars and suggests an exponential‐like growth of *k* with *η*. This could be related to the diversified plant adaptation as a function of the distance from the water table.

The decay rate *α*(*k*) varies within a narrow range of order 1 (Figure 11b), and it decreases with the inundation probability (bar 1) or just after a peak value at *P*_*I*_ = 0.4 (bar 2). Physically speaking, *α*(*k*) is the rate of biomass reduction after flood events (Camporeale & Ridolfi, 2006). The decreasing trend in Figure 11b suggests that the farther from the main channel, the faster vegetation decays once major flooding happens. This result originates from the simplified approach of the model, which include different tree species into a single vegetation variable. The riparian zone is in reality commonly characterized by species zonation (Francis, 2007; Karrenberg et al., 2003; Stromberg et al., 1993), and as we verified during the field survey, also the Cinca riparian area shows precise patterns of different plant species. Willows and young poplars colonize sites close to the water, whereas the adult poplars are usually found in more elevated, drier, and less fluvially active areas. This zonation can be easily related to the probability of inundation, since the two considered tree species have different root resistance. Willows perform a great uprooting resistance (equivalent to low *α*) and therefore are able to colonize the sites that are more subjected to flood disturbance (high *P*_*I*_). Instead, poplars are characterized by a lower resistance (i.e., high *α*), and although young poplars can establish close to the water, the presence of mature trees is usually favored by soil aggradation so that they can be found on more elevated and stabilized zones. Therefore, the smooth variation of *α*(*k*) versus *P*_*I*_ (Figure 11b) probably mirrors the gradual transition between areas with prevailing willows to areas with prevailing poplars (Karrenberg et al., 2003).

Concerning the local carrying capacity *β*, the two bars show similar behaviors (Figures 11c and 11d). *β* has a maximum for *P*_*I*_ = 0.7–0.8, while decrease for extremely high or low values of *P*_*I*_. The presence of a maximum is a clue of optimal conditions of soil moisture and, therefore, the existence of an optimal water table depth. The trend we found for the calibrated carrying capacity versus the elevation (circles in Figure 11e) is consistent with some previous field observations (Francis, 2007; Pasquale et al., 2012; Shafroth et al., 2000), wherein the deepening of the phreatic surface was considered as the major controlling factor for the maximum plant size. A comparison between the present results and the parabolic function recommended by Phipps (1979) and Pearlstine et al. (1985) is also reported in Figure 11e. It is evident that our field‐based findings suggest that *β* is properly fitted by a skewed curve rather than a symmetric parabola. The results on *β* indicate species zonation as well. The larger values of *β* close to the main channel in fact reveals the presence of willows, which develop larger above‐ground biomass than poplars (Karrenberg et al., 2003). This result is again in agreement with the observations made during the field survey.

Since the two bars are morphologically different, they are subjected to different hydraulic conditions. In particular, high flows spread more uniformly on bar 2 than in bar 1. This has an effect on the vegetation response, which appears very sensitive to small changes in hydromorphology. From the calibration, it turns out that *k*, *α*, and *β* have similar qualitative trends (Figure 11), but the small differences in their numerical values preclude the interchangeability of calibrated values.

**Figure 12:**
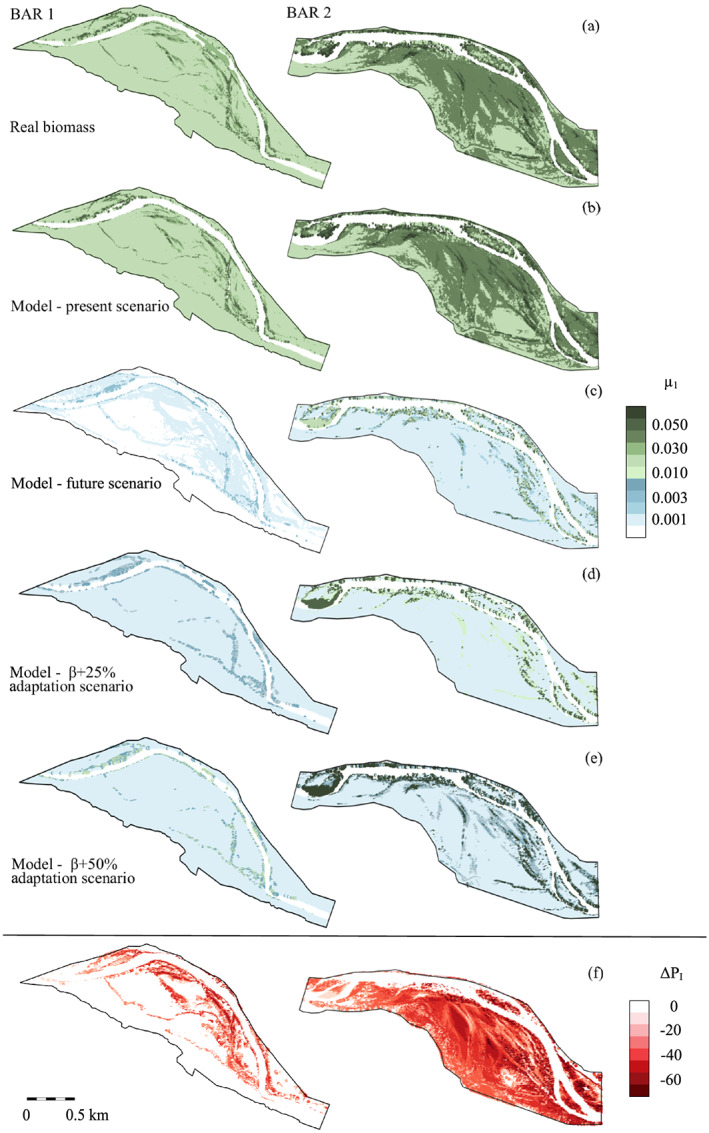
Visual comparison between (a) real and (b) computed average biomass *μ*_1_ for the two bars. Computed average biomass for a 40% flow rate reduction without (c) adaptation, and assuming a partial adaptation to the modified hydrological forcing by increasing the carrying capacity *β* of (d) 25% and (e) 50%, respectively. (f) Distribution of the percentage difference of the probability of inundation Δ*P*_*I*_ between the current and future scenarios, with negative values indicating *P*_*I*_ reduction.

We finally emphasize the excellent matching between the two maps for the spatial distributions of the average biomass obtained from real data and the calibrated model, respectively (Figures 12a and 12b). Numerically speaking, the overall percentage absolute difference between panels (a) and (b) amounts to 9.3% and 3.3%, for the bar 1 and 2, respectively. This result shows that the calibration of the model parameters on *P*_*I*_‐classified real data provides the sufficient information to reconstruct the biogeography of the riparian zone correctly, despite of the simplicity of the model.

### 7.2 Impact Modeling

A LIM is a regional version of the *global* impact model (GIM), which supports strategies for climate adaptation and mitigation by accounting for the effects of climate change at the global scale (Giuntoli et al., 2015). LIMs are focused on smaller length scales in order to investigate the environmental response to climate change. We use the calibrated model CR06 as a LIM with the aim of forecasting the future distribution of riparian biomass in a climate‐change scenario.

It is well known that future climate projections indicate an increase in the mean global temperature with remarkable effects on the hydrological regimes worldwide (Dankers & Hiederer, 2008; Pachauri et al., 2014). In the Northern Spain and in the Ebro River basin, the annual precipitation is expected to decrease up to 30%. This, coupled with the increased evapotranspiration due to higher temperatures, will potentially lead to a 40% reduction in the mean annual daily flow rate by the end of 2100 (Alfieri et al., 2015).

According to this projection, we artificially modified the log normal pdf of the daily flow rates by reducing the mean value of the 40% and increasing the coefficient of variation (the standard deviation/mean ratio) of 15% (Alfieri et al., 2015). This new pdf of flow rate is shown by a dash‐dot line in Figure 5a. It is worth noticing that, as the mean flow decreases, we may expect that the morphological activity of the riparian area will be even further reduced (in agreement with the model hypothesis of a steady morphology). Further, we remark that the solutions reported for the future scenario refer to the statistically steady‐state condition for the biomass, that is, successive to a possible a transitory phase.

We replicated the hydrodynamic modeling (section 6.1) in order to assess the new scenario of the *P*_*I*_ distribution. The percentage difference Δ*P*_*I*_ between the two scenarios is shown in Figure 12e, indicating that the strong reduction of the mean flow leads to an overall drop of the probability of inundation (i.e., negative Δ*P*_*I*_ values). After the computation of the new hydrological statistics, the model was ready to provide a forecast of the vegetation evolution in the study site, since the biological parameters *k* and *β* were already calibrated. Figure 12c shows the mean biomass expected in the future scenario. A moderate variation in the hydrological condition induces a dramatic change in the riparian biogeography. This is indicative of the high sensitivity of the riparian zone to external forcing, where the negative effects of a lower mean flow exceed the beneficial reduction of flooding magnitude. The vegetation strongly reduces in response to an excessive deepening of the phreatic water table, which we indeed identified as the controlling factor in the Cinca River. In this future scenario, the biomass is high only in those areas where *P*_*I*_ is equal to 0.8–0.9 (close to the water) and then rapidly drops, mirroring again the strong correlation between favorable soil moisture conditions and proximity to the water table. In addition, because of the low discharges, the river slightly narrows, and some new sites are available for vegetation colonization (see for instance the upstream part of bar 2).

The above projection shows that the extension of the highly vegetated areas decrease in response to the expected change in the hydrological forcing. This implies an overall reduction of the habitats and a loss in biodiversity. However, this simulation overlooks a crucial feature of natural systems, namely, adaptability. The gradual nature of the hydrological change may allow the riparian vegetation to adopt different adaption strategies. This might occur through morphological adaptation (Kozlowski, 1984) of the autochthonous plants or with the spread and colonization of invasive species (Tickner et al., 2001; Vesipa et al., 2017).

In light of the above remarks, we modified the biological parameters in order to mimic plant adaptation in the present modeling framework. To this aim, we arbitrarily increased the calibrated carrying capacity values reported in Figure 11c by 25% and 50%. The resulting biomass density is reported in Figures 12d and 12e, respectively. The *β* increment of 25% enables vegetation to survive in the driest areas of the study site (the white areas in Figure 12c) and promotes the growth of vegetated plots where *P*_*I*_ = 0.7–0.9. These positive effects are further enhanced if the carrying capacity increment is equal to 50% (Figure 12e). In a nutshell, these numerical experiments could be used to investigate the resilience of riparian ecosystems to changing environmental forcing.

## 8 Conclusions

Riparian environments are extremely biodiverse and delicate ecosystems, which are undergoing an intense human‐ and climate‐induced pressure. The need for conservation and restoration plans is becoming compelling and theoretical and quantitative ecomorphodynamic models could provide a useful support.

In this paper, we have proposed an integrated methodology to calibrate a minimalist stochastic model for riparian vegetation dynamics onto real data. A major advantage of the model is that only few biological parameters need to be set for the intrinsic features of vegetation dynamics. Particularly, when the model is applied to investigate the steady‐state solutions of the biomass distribution, the required biological parameters reduce to two, namely, the local carrying capacity *β* and the flood‐induced decay rate of vegetation *α*(*k*). Once calibrated, the model can be used to assess the present and future vegetation cover in the study site.

Regarding the biological parameters (Figure 11), the field‐based calibration shows a topographic dependence of the flood‐induced decay rate *α*(*k*), which reflects different plant adaptations and species zonation. This result also suggests that the decay process should be considered spatially dependent in theoretical models for the vegetation dynamics. The carrying capacity *β* is also influenced by species zonation and depends on the depth of the phreatic surface and the related soil moisture conditions. The results also show that the commonly suggested parabolic shape for *β* is an idealization, which significantly differs from real trends, according to which *β* is better reproduced by a right‐skewed curve. Overall, further applications of the methodology could broaden the data set of calibrated *β* and *α*(*k*) and improve the understanding of their relationship with the topographical and hydrodynamical statistics.

The calibrated model is then shown to well reproduce the actual biogeography of the Cinca riparian zone. The overall percentage absolute difference between the real and the computed biomass amounts to 9.3% and 3.3% for two different vegetated bars. We have also illustrated a possible application of the calibrated model as a LIM. To this aim, we evaluated the effects that a climate‐change scenario can induce on the riparian environment of the Cinca River, where a reduction of the mean water flow is expected (Alfieri et al., 2015). The model foresees an overall reduction of vegetation cover, which almost disappears where the phreatic surface becomes too deep. The model thus demonstrates that riparian vegetation is highly sensitive to changes in external forcing, a feature that is particularly important when considering short‐term dramatic changes that do not allow for adaptation strategies (Lombardi et al., 2017). Nevertheless, for more gradual hydrological variations, it is possible that the riparian ecosystem adapts through morphological transformation of the autochthonous plant species or by the invasion of more resistant allochthonous species. We briefly modeled these aspects by an arbitrary modification of the biological parameters. The results show some resilience of the riparian biomass just in those areas where favorable conditions to plant growth, that is, reduced floods and sufficient soil moisture, persist.

The methodology and the calibration procedures here presented rely on a novel integration of different tools of river science, ranging from theoretical and numerical modeling, to stochastic processes, geomatics, and field activities. The conceptual scheme is summarized in Figure 1. Nevertheless, we stress out that different *modus‐operandi* can be adopted at every step of the procedure. For example, we calibrated and used the stochastic model by Camporeale and Ridolfi (2006), but the methodology could benefit from more advanced models (Camporeale & Ridolfi, 2010; Muneepeerakul et al., 2007; Tealdi et al., 2013; Vesipa et al., 2015).

We decided to adopt the Camporeale and Ridolfi (2006) model since it catches relevant aspects of riparian processes, although overlooking the morphological activity of the study site. The model enabled us to highlight the central role of the distance from the water surface and the inundation probability, which together summarize the local hydrological and morphological conditions, in driving the development and decay of riparian vegetation. When morphological changes do not occur, the water‐induced zonation remains constant, and the model satisfactorily suits reality. However, the morphological activity of riparian areas generally induces changes in the inundation probability and in the local moisture, so promoting new hydrology‐vegetation feedback. It is therefore our intention to explore the temporal dynamics of the riparian environments in future analyses by using not only a single set of LiDAR data but also a LiDAR time series from which a temporal trend of vegetation biomass and topography can be inferred. This would also allow to study the temporal evolution of the calibrated biological parameters, with a deeper understanding of their variability and adaptation strategies.

We finally remark that, in our framework, the river morphology is considered as static. The riverbed of the Cinca River has been undergoing degradation since 1969, when the El Grado Reservoir was built. Indeed, a quick view to the timelapse on free database of Google Earth Engine imaging shows that the two bars focus of the analysis have not moved for the last 30 years (Figure 4). Riverbed degradation and future lower flow rates reinforce the validity of our assumption of a weak dynamic activity of the river morphology. Yet extreme formative flooding events may occur and affect the overall river morphology. Our results on the forthcoming vegetation dynamics have then to be thought on a wider perspective than just on the two bars focus of the analysis. In a more general parlance, we may expect a decrease in the vegetation cover far from the river bank, because of the increasing distance from the phreatic surface, and an increase of the vegetation biomass close to the river banks, unless exceptionally strong floods may alter this picture. In this way, the calibrated model appears to be functional to the understanding of riparian ecosystems dynamics and a promising tool for management strategies of future scenarios.

## Data Availability

Data sets for this research are available in the following in‐text data citation references: LiDAR data were provided by the Spanish National Center of Geographic Information (IGN, 2015) (open access); hydrological data were provided by the Ebro river basin authority (CHE, 2010) (upon request); field data were collected by the authors and are available online (Latella et al., 2020) (open access).

## Appendix A LiDAR Data Processing

### A.1

We processed and visualized LiDAR data through the FUSION/LDV software package. In the following, an insight into the basic operations for the procedure is provided. Further specifications for each function can be found in the FUSION/LDV handbook (McGaughey, 2016).

First, the available LiDAR data sets were decompressed by running the *Lda2Las* function, while the *Catalog* function returned a grayscale intensity image associated to the data. Each of the available data set covered a square area with sides 2 km long. The grayscale images were imported in GIS environment and a shapefile circumscribing the study site was then defined. This selection reduced the computational effort and allowed to fasten the subsequent implementation of the *GroundFilter* and *GridSurfaceCreate*functions.

*GroundFilter* consists of the iterative implementation of a filtering algorithm to return an intermediate surface between the ground and the canopy. The algorithm has been adapted from Kraus and Pfeifer (1998) by FUSION/LDV developers, and it comprises a system of three equations:

(A1) $$f_{i}=\left\{\begin{array}{ll} 1 & \;v_{i}\leq l_{1}\\ \frac{1}{1+a{\left(v_{i}-l_{1}\right)}^{b}} & \;l_{1}<v_{i}\leq (l_{1}+l_{2})\\ 0 & \;(l_{1}+l_{2})<v_{i} \end{array}.\right)$$

At each iteration, Equation A1 defines an intermediate surface so that every *i* point is located at a certain vertical distance *v*_*i*_ from the surface, with ground and canopy points expected to be below or above it, respectively. In Equation A1, *l*_1_ indicates the threshold distance below which the weight function *f*_*i*_ is 1, indicating points belonging to the intermediate surface. Instead, *l*_2_ defines the upper limit for points to have an influence on the ground surface. All the points farther than *l*_1_+*l*_2_ from the surface have a weight *f*_*i*_ equal to zero. Finally, the *a* and *b* coefficients determine the steepness of the weighting function. At the end of the iterations, this algorithm returns the bare earth surface of the analyzed area.

In our case, *l*_1_ and *l*_2_ were set respectively equal to −1 and 2.0, while the tolerance was fixed as 0.01. Although various numbers of iterations were tried (from 10 to 1,000), no relevant differences were noticed in the outcomes. Thus, we set 100 iterations to balance precision with computational time. The output surface was then used as the input for the *GridSurfaceCreate* function, which returns the DTM of the investigated area. In turn, this DTM became the input topography for the two‐dimensional hydrodynamic modeling (see Appendix B).

Finally, we used the *GridMetrics* function to extract statistical information about vegetation. This function splits the covered area according to a square‐cell grid (we set cells having 2‐m sides) and computes descriptive statistic parameters referring to vegetation height and coverage. Subsequently, we coupled these statistics to the allometric relationships derived from the field survey in order to compute vegetation biomass (see section 5.4).

## Appendix B Delft3D‐FM Setup for the Hydrodynamic Modeling

### B.1

The software Delft3D‐FM was chosen for the opportunity of applying two of its modules, namely the innovative *flexible‐grid generator*, and the *trachytope* functionalities. The flexible grid represents the evolution of traditional curvilinear grid, as it adds to the curvilinear cells, which well fit the river channels, and triangular ones, which allow for a coarser cover of the floodplain and the fitting of eventual obstacles (e.g., bridge piers). The trachytope module enables the user to delimit the areas where the roughness is homogeneous and to associate to each area a roughness value (or a formula to compute it). Thus, this module grants the inclusion of the vegetation contribution to the local roughness. In order to get the water depth of each cell for a given flow rate, we adopted a two‐dimensional depth‐averaged approach in order to reduce the computational costs of the analysis.

Table B1 shows the parameter setting of the numerical model, which was similar for both the bars. The DTM that had been previously obtained by the processing of LiDAR data with FUSION/LDV was used to define the topography. The flexible mesh was created according to the physical features of the investigated bars, setting 10‐m side curvilinear cells for the main channel and triangular cells for the remaining domain. The triangular cells were slightly coarser for an increasing distance from the main channel. The roughness was set by discretizing the domain in trachytopes. A Manning coefficient, derived by field data processing, was used for the main channel and the bare ground (Table 2). The vegetated areas were grouped according to homogeneous tree heights and stem densities and then associated to the Barneveld's formula in Delft3D. This formula was first proposed by Klopstra et al. (1997) and then modified to include also the contribution of the bare bed to the total roughness. According to this formula, the roughness *C*_*t*_ in areas with emerged vegetation is a function of the drag coefficient *C*_*d*_, the number of stem per square meter *λ*, the stem diameter *D*, the water level *h*, and the bare bed roughness *C*_*b*_:

(B1) $$\frac{1}{C_{t}^{2}}\;=\;\frac{C_{d}\lambda Dh}{2g}+\frac{1}{{C_{b}}^{2}}.$$

The boundary conditions were set as upstream flow rates and downstream water levels, according to the available data. As our aim was to investigate the relationship between the inflow and the local water level, we arbitrarily chose 19 reference values from the pdf of flow rates, sampling them every 5th percentile, and we carried out one simulation for each of them after having posed it as upstream boundary condition.

**Table B1:** Parameter Setting for Delft3D‐FM

Parameter	Value	Unit	Notes
Maximum Courant number	0.7	‐	Based on software guidelines
Modeled area (width × length)	850 × 3,700	m	To cover the investigated bars
Average triangular cell size (width × height)	9 × 9	m	Resolution‐efficiency compromise
Average curvilinear cell size (width × height)	4.5 × 9	m	Resolution‐efficiency compromise
Average curvilinear aspect ratio	1:2	m	Based on software guidelines
Median diameter (d_50_)	0.11	m	From collected field data
Manning coefficient bare ground	0.03	‐	From collected field data
Trachytope formula for bare areas	53	‐	Manning value
Trachytope formula for vegetated areas	152	‐	Barnevelds formula (see..)

## Appendix C Nomenclature

### C.1

**Table C1:** Symbols Used in the Paper and the Relative Definition

Symbol	Definition	Symbol	Definition
*α*	Dimensionless decay rate	*d*	Sediment diameter (m)
*β*	Dimensionless local carrying capacity	*f*_*i*_	Kraus weight function
*δ* (*δ*^∗^)	Dimensionless (dimensional) phreatic surface depth	*g*	Gravitational acceleration (m s^−2^)
*ζ* (*ζ*^∗^)	Dimensionless (dimensional) phreatic surface level	*H*	Tree height (m)
*η*, *η*^∗^	Dimensionless (dimensional) topographic level	*h* (*h*^∗^)	Dimensionless (dimensional) water level
*λ*	Tree density (m^−2^)	*k*	Tree species‐dependent decay coefficient
*μ*_1_	Average vegetation biomass	*m*,*n*,*p*	Tree species‐dependent parameters
*ν*	Dimensionless vegetation biomass	*N*	Normalization constant
*ρ*	Green wood density (Mg m^−2^)	*n*_*m*_	Manning coefficient
*τ* (*τ*)	Dimensionless (dimensional) integrale scale	*P*_*I*_	Probability of inundation
*τ*_*I*_ (*τ*_*E*_)	Duration of inundation (exposure)	*Q*	Daily flow rate (m^3^ s^−1^)
*ω*	Rate of vegetation growth (d^−1^)	*S*	Number sediment sample class
*a*, *b*, *l*_1_, *l*_2_	Coefficient for the Kraus and Pfeifer equation	*s*	Weight sediment sample class
*C*_*b*_	Hydraulic bare bed roughness	*V*	Above‐ground tree biomass (Mg m^−2^)
*C*_*d*_	Drag coefficient	v	Vertical distance from the surface (m)
*C*_*t*_	Hydraulic roughness	*w* (*w*^∗^)	Dimensionless (dimensional) transect width
*D*	Tree diameter at breast height (m)	*x* (*x*^∗^)	Dimensionless (dimensional) horizontal coordinate
